# One-year Oxford knee scores should be used in preference to 6-month scores when assessing the outcome of total knee arthroplasty

**DOI:** 10.1186/s43019-020-00060-5

**Published:** 2020-08-28

**Authors:** N. D. Clement, N. Ng, D. MacDonald, C. E. H. Scott, C. R. Howie

**Affiliations:** 1 Department of Orthopaedics, The Royal Infirmary of Edinburgh, 51 Little France Cres, Edinburgh, EH16 4SA UK; 2 Department of Orthopaedics, University of Edinburgh, Royal Infirmary of Edinburgh, 51 Little France Cres, Edinburgh, EH16 4SA UK

**Keywords:** Change, Difference, 6 Months, 12 Months, Total knee arthroplasty, Outcome, Oxford knee score

## Abstract

**Purpose:**

The primary aim of this study was to assess whether there was a clinically significant difference in the mean Oxford knee score (OKS) between 6 and 12 months after total knee arthroplasty (TKA). The secondary aim was to identify variables associated with a clinically significant change in the OKS between 6 and 12 months.

**Methods:**

A retrospective cohort study was undertaken using an established arthroplasty database of 1574 primary TKA procedures. Patient demographics, body mass index (BMI), comorbidities, OKS and EuroQoL 5-domain (EQ-5D) score were collected preoperatively and at 6 and 12 months postoperatively. A clinically significant change in the OKS was defined as 5 points or more.

**Results:**

There was a 1.1-point increase in the OKS between 6 and 12 months postoperatively, which was statistically significant (95% confidence (CI) 0.8–1.3, *p* < 0.0001). There were 381 (24.2%) patients who had a clinically significant improvement in their OKS from 6 to 12 months. After adjusting for confounding, patients with a lower BMI (*p* = 0.028), without diabetes mellitus (*p* < 0.001), a better preoperative OKS (*p* < 0.001) or a worse 6-month OKS (*p* < 0.001) were more likely to have a clinically significant improvement. A 6-month OKS < 36 points was a reliable predictor of a clinically significant improvement in the 6-month to 12-month OKS (area under the curve 0.73, 95% CI 0.70–0.75, *p* < 0.001).

**Conclusion:**

Overall, there was no clinically significant change in the OKS from 6 to 12 months; however, a clinically significant improvement was observed in approximately a quarter of patients and was more likely in those scoring less than 36 points at 6 months. Level of evidence: retrospective diagnostic study, level III.

## Introduction

Patient reported outcome measures (PROMs) are valuable tools that are commonly used to assess the outcome of total knee arthroplasty (TKA) [1]. The Oxford knee score (OKS) [2] is a joint-specific PROM and is the outcome measure of choice in England and Wales to evaluate the functional outcome of TKA [3]. The OKS is a validated assessment tool and has been shown to be reliable, reproducible and capable of measuring a clinical change after TKA [2, 4]. A greater improvement in the OKS has been demonstrated to correlate with an increased rate of patient satisfaction with the outcome of their TKA [5, 6].

The optimal timepoint at which the OKS should be assessed after TKA is not clear. A systematic review by Browne et al. [7] recommended that the 12-month OKS be used rather than the 6-month OKS as there was a further 2-point improvement from 6 to 12 months postoperatively. The OKS appears to peak at 12 months and remains stable into the mid to long term [8–10]. If the OKS increases further between 6 and 12 months, this may lead to differing conclusions when assessing the same cohort of patients [11, 12]. Although there may be a 2-point improvement in the OKS from 6 to 12 months after TKA, it is not known whether this is statistically and clinically significant or which patients are more likely to experience OKS improvement.

The primary aim of this study was to assess the whether there was a clinically significant difference in the mean OKS between 6 and 12 months after TKA. The secondary aims were to (1) identify predictors of change in the 6-month to 12-month OKS, (2) assess the proportion of patients that had a clinically significant change in their OKS between 6 and 12 months and (3) identify independent variables associated with a clinically significant change. The null hypothesis was that no clinically significant change occurs in the OKS between 6 and 12 months.

## Patients and methods

Patients for this study were identified retrospectively from a prospectively compiled arthroplasty database held at the study centre. During a 57-month period (January 2013 to September 2017) 2550 TKA were performed at the study centre and patients were asked to complete a preoperative patient questionnaire at their preassessment clinic. Medical staff were on hand to assist and answer any questions. The patient demographics, body mass index (BMI) and comorbidities were recorded at the preoperative assessment. Categories of comorbidity included were myocardial infarction (MI), heart failure, peripheral vascular disease (PVD), stroke, dementia, chronic obstructive airways disease (COPD), diabetes mellitus, back pain and pain in other joints, which were recorded as dichotomous variables. Patients who did not complete their OKS assessments preoperatively or at 6 and 12 months were also excluded from analysis. There were 976 (38.3%) patients excluded as they did not complete their preoperative OKS and their 6-month or 12-month OKS. The study cohort consisted of 1574 event-linked OKS. Patients excluded were slightly younger (70.3 versus 69.1 years, difference 1.2, 95% confidence intervals (CI) 0.5–1.9, *p* = 0.002), but there was no difference in gender (*p* = 0.13) or BMI (*p* = 0.18). Patients were subsequently contacted at 6 and 12 months by post to complete functional assessment questionnaires. Patients were able to contact the orthopaedic team should they have had any questions at these assessment times.

The OKS was recorded preoperatively and at 6 and 12 months postoperatively. The OKS is derived from 12 questions assessed on a Likert scale with values from 0 to 4; a summative score is then calculated where 48 is the best possible score (least symptomatic) and 0 is the worst possible score (most symptomatic) [2]. The minimal clinically important difference (MCID) for the OKS is 5 points, which is thought to represent a clinical difference between two groups of patients [10].

The EuroQoL (EQ) general health questionnaire, which evaluates five domains (5D) to assess mobility, self-care, usual activities, pain/discomfort and anxiety/depression, was recorded preoperatively and at 6 months postoperatively [13]. The 3-level (3-L) version of the EuroQoL questionnaire was used, with the responses to the five domains being recorded at 3 levels of severity (no problems; slight problems; moderate, severe or unable/extreme problems). This index is on a scale of − 0.594 to 1, where 1 represents perfect health, and less than 0 represents a state worse than death.

During the study period two type of implants were used: Triathlon (Stryker, Marwah, NJ, USA) and the PFC Sigma (DePuy, Johnson & Johnson Professional Inc., Raynham, MA, USA). A measured resection technique was used. All patients were reviewed at a preassessment clinic. A standardised rehabilitation protocol was used for all patients, with active mobilisation on the first postoperative day. Patients were then reviewed at 6 weeks, 6 months and 12 months postoperatively.

Statistical analysis was performed using the Statistical Package for Social Sciences version 17.0 (SPSS Inc., Chicago, IL, USA). Student’s paired or unpaired *t* test was used to compare linear variables between groups. Dichotomous variables were assessed using the chi square test. Pearson’s correlation was used to assess the relationship between linear variables. Multivariate linear regression analysis was used to identify independent predictors associated with change in the OKS from 6 to 12 months. Simple linear regression analysis was used to identify change in the 6–12-month OKS according to the 6-month OKS. Multivariate logistic regression analysis was used to identify independent predictors associated with change greater than the MCID in the OKS from 6 to 12 months. Receiver operating characteristic (ROC) curve analysis was used to identify a threshold (cut points) in scalar variables (preoperative and 6 months OKS and BMI) that were independently associated with a change equal to or greater than the MCID between 6 and 12 months. The area under the curve (AUC) ranges from 0.5, indicating a test with no accuracy, to 1.0 where the test is perfectly accurate by identifying all satisfied patients. A *p* value <0.05 was defined as significant. A post hoc power calculation was performed using the OKS (primary outcome measure) and the MCID of 5 points  [10] with standard deviation (SD) of 10 points and alpha of 0.05, and using a two-way analysis, power of 100% was achieved in this study cohort (*n* = 1574).

Ethical approval was obtained from the regional ethics committee (Research Ethics Committee, South East Scotland Research Ethics Service, Scotland (11/AL/007)]) for analysis and publication of the presented data.

## Results

There were 1574 TKA performed during the study period with complete preoperative and postoperative data that met the inclusion criteria. There were 711 male patients and 863 female patients, with a mean age of 70.3 (SD 9.0, range 23–93) years. The mean preoperative OKS was 21.3 (SD 7.8), which by 6 months postoperatively had increased to a mean of 35.2 (SD 9.0); the 13.9-point improvement was significant (95% confidence interval (CI) 13.4–14.4, *p* < 0.0001 paired *t* test). At 12 months postoperatively the OKS increased further to 36.2 (SD 9.6) and the 15.0-point improvement was significant (95% CI 14.5–15.4, *p* < 0.0001 paired *t* test).

### Primary outcome

There was a 1.1-point increase (improvement) in the OKS between 6 and 12 months postoperatively, which was statistically significant (95% CI 0.8–1.3, *p* < 0.0001 paired *t* test). However, this 1.1-point increase was not greater than the MCID.

### Secondary outcomes

#### Predictors of change in OKS from 6 to 12 months

**Fig. 1:**
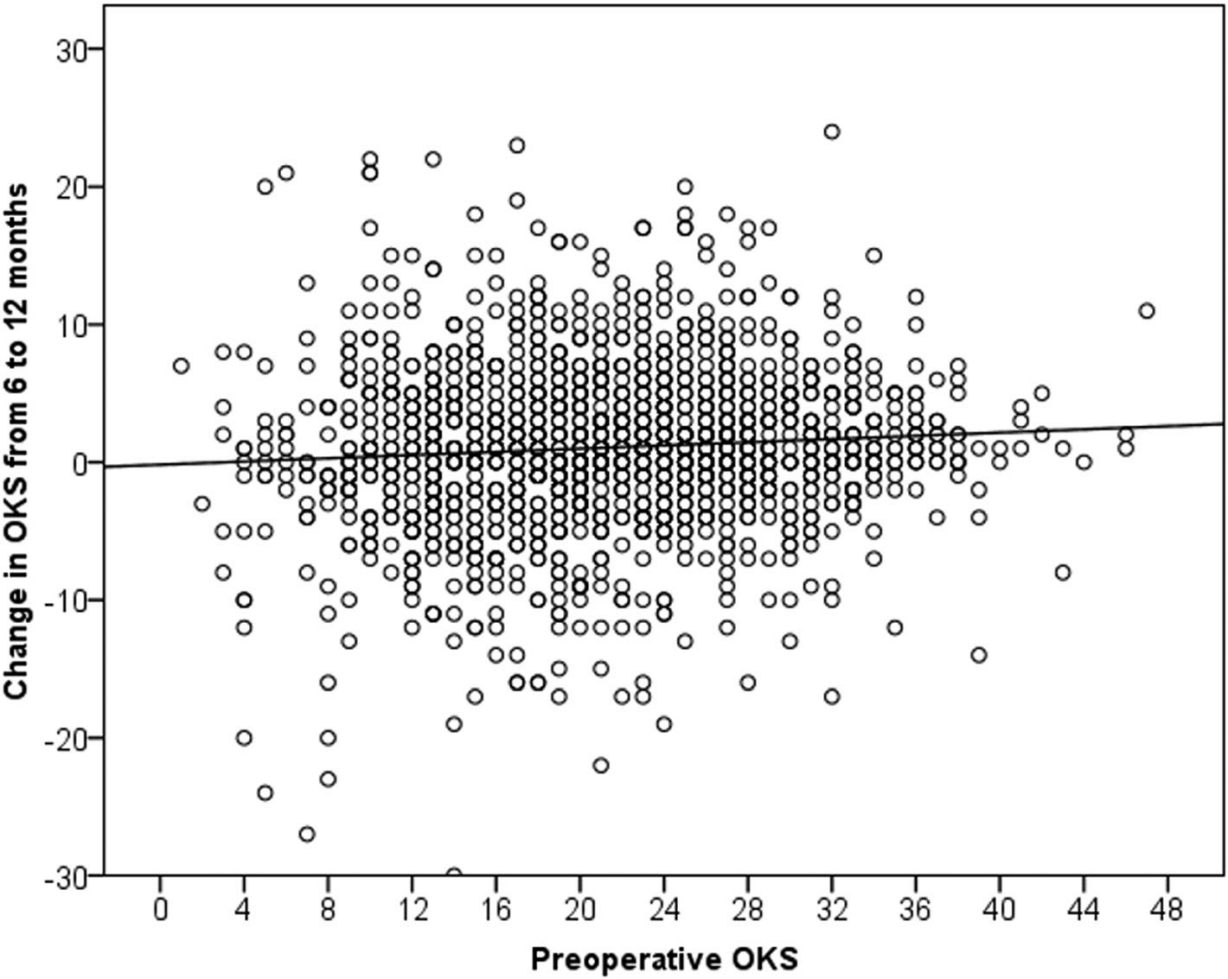
Scatter plot for the study cohort according to the preoperative Oxford knee score (OKS) and change in the OKS from 6 to 12 months after total knee arthroplasty (TKA). The black line represents a linear line of best fit (*r* = 0.08 Pearson correlation)

**Fig. 2:**
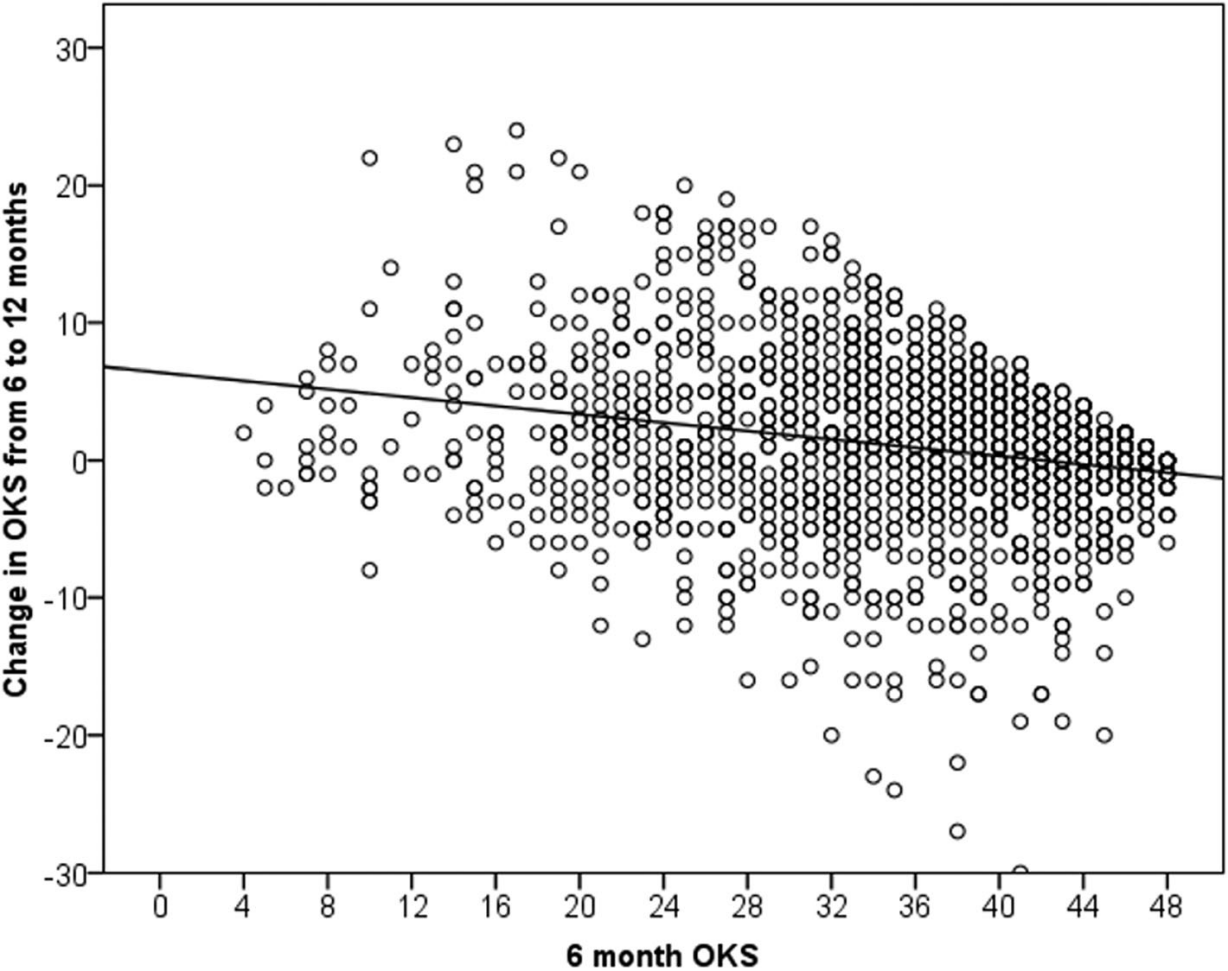
Scatter plot for the study cohort according to the 6-month Oxford knee score (OKS) and change in the OKS from 6 to 12 months after total knee arthroplasty (TKA). The black line represents a linear line of best fit (*r* = −0.23 Pearson correlation)

Patients without diabetes mellitus and patients with a better (greater) preoperative OKS, a worse 6-month OKS and a better EQ-5D at 6 months were significantly more likely to have a greater improvement in the OKS from 6 to 12 months on both unadjusted (Table 1) and multivariate (Table 2) analysis. None of the other comorbidities, apart from diabetes mellitus, were associated with change in the OKS from 6 to 12 months (Table 1). There was a trend toward lower BMI being associated with a greater change in the 6-month to 12-month OKS (Table 1), which was significant after adjusting for confounding (Table 2). A better preoperative OKS resulted in a greater improvement in the OKS from 6 to 12 months (Fig. 1), whereas in contrast, a worse OKS at 6 months resulted in a greater improvement from 6 to 12 months (Fig. 2). Simple linear regression analysis demonstrated that the 6-month OKS influenced the change in the 6-month to 12-month OKS by up to 6.4 (95% CI 5.3–7.6, *p* < 0.001) points, which exceeded the MCID. Using this same regression model, it would be possible to calculate the change in the OKS between 6 and 12 months using the 6-month OKS: change between 6 and 12 months = 6.4 – (6 months OKS × 0.152).

**Table 1:** Predictors of change in the OKS between 6 and 12 months after TKA

Demographic	Descriptive	Number	Mean (SD)	Difference/ correlation	95% CI	***P*** value*
**Gender**	Male	711	1.1 (6.3)	Diff = 0.0	−0.6 to 0.6	0.98
	Female	863	1.1 (5.6)			
**Age**			–	*r* = −0.04		0.16**
**BMI**			–	*r* = −0.05		0.054**
**Comorbidity**	Not present			Reference***		
	MI	65	0.3 (7.4)	Diff = 0.8	−0.6 to 2.3	0.27
	Heart Failure	15	3.1 (9.6)	Diff = 2.0	−1.0 to 5.0	0.18
	PVD	31	1.1 (8.1)	Diff = 0.1	−2.0 to 2.2	0.93
	Stroke	10	−1.8 (8.1)	Diff = 3.0	−0.7 to 6.6	0.11
	COPD	57	0.8 (6.6)	Diff = 0.4	−1.2 to 1.9	0.66
	Diabetes	189	0.3 (6.6)	Diff = −1.0	−1.9 to −0.1	**0.04**
	Back pain	590	1.2 (6.4)	Diff = 0.2	−0.5 to 0.8	0.62
	Joint pain	1041	1.0 (5.9)	Diff = 0.4	−0.3 to 1.1	0.22
**Functional measures**						
**OKS**	Preoperative	1574	–	*r* = 0.08		0.002**
	6 months	1574	–	*r* = −0.23		< 0.001**
**EQ 5D**	Preoperative	1570	–	*r* = 0.04		0.14**
	6 months	1567	–	*r* = −0.11		< 0.001**

*TKA* total knee arthroplasty, *OKS* Oxford Knee Score, *EQ 5D* EuroQoL 5-Dimension questionnaire, *BMI* body mass index; *MI* myocardial infarction, *PVD* peripheral vascular disease, *COPD* chronic obstructive pulmonary disorder, *CI* confidence interval, *Diff* difference

*Unpaired *t* test unless otherwise stated. **Pearson correlation. ***Difference relative to a patient without the stated comorbidity

Bold indicates statistical significance

**Table 2:** Independent predictors associated with change in the OKS from 6 to 12 months after TKA

Demographic	Descriptive	Change in OKS	95% CI	***P*** value
**BMI**		−0.07	− 0.13 to − 0.02	0.011
**Comorbidity**	Not present	Reference		
	Diabetes mellitus	−1.2	−2.07 to −0.30	< 0.001
**Functional measures**				
**OKS**	Preoperative	0.14	0.10 to 0.18	< 0.001
	6 Months	−0.31	−0.37 to − 0.26	< 0.001
**EQ 5D**	6 Months	4.80	2.66 to 6.94	< 0.001

Linear regression analysis: all significant (*p* < 0.05) variables or variables with a trend towards statistical significance (*p* < 0.1) were entered into the model. The *R*^2^ value of the model was 0.12

*TKA* total knee arthroplasty, *OKS* Oxford Knee Score; *EQ 5D* EuroQoL 5-Dimension questionnaire, *BMI* body mass index, *CI* confidence interval

#### Change greater than the MCID in the OKS between 6 and 12 months

**Fig. 3:**
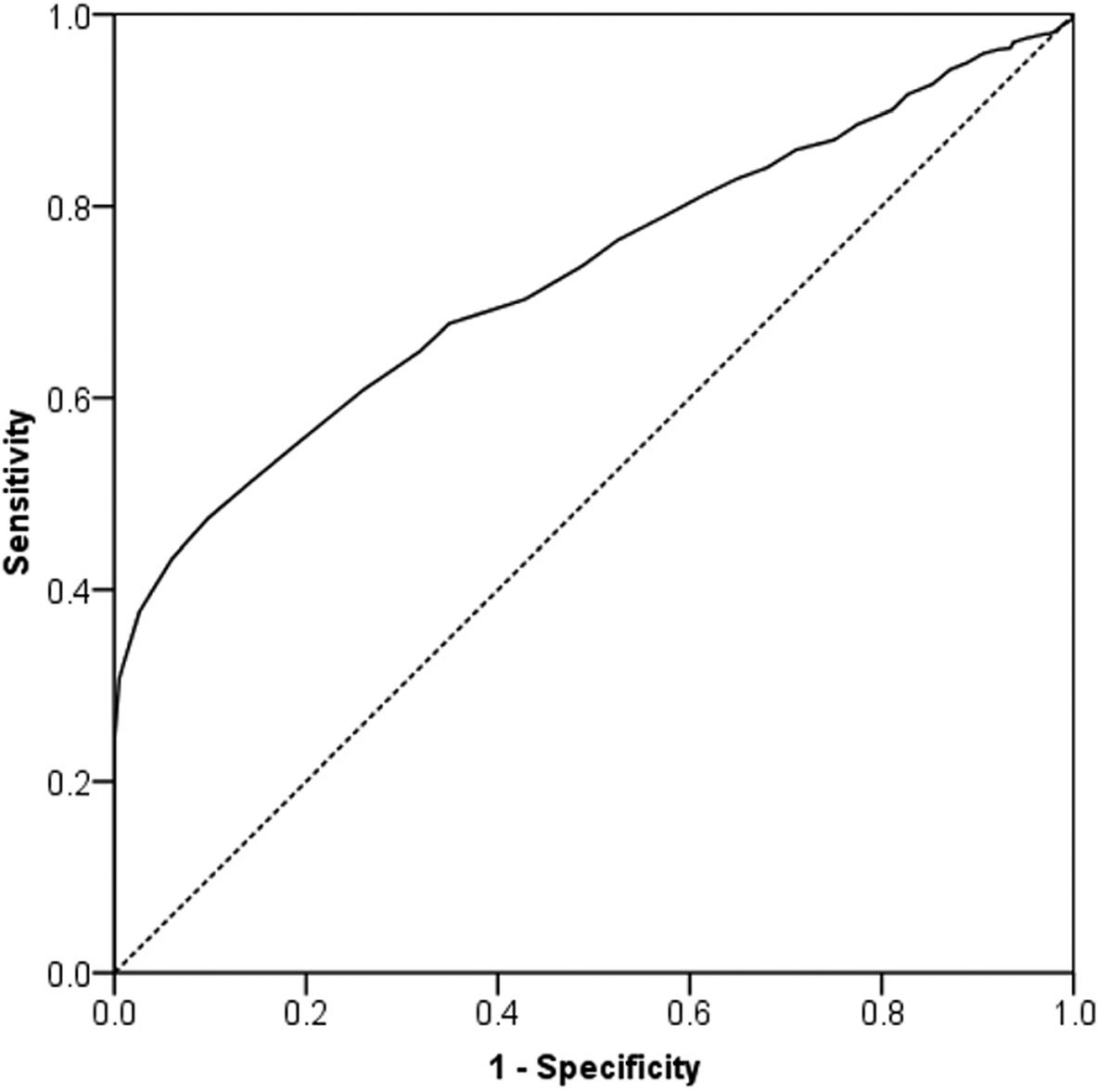
Receiver operating characteristic (ROC) curve for predicting a 5-point or greater (minimal clinically important difference (MCID)) improvement in the Oxford knee score (OKS) from 6 to 12 months according to the 6-month OKS (black line). Dashed line is a reference line for no predictive ability

**Fig. 4:**
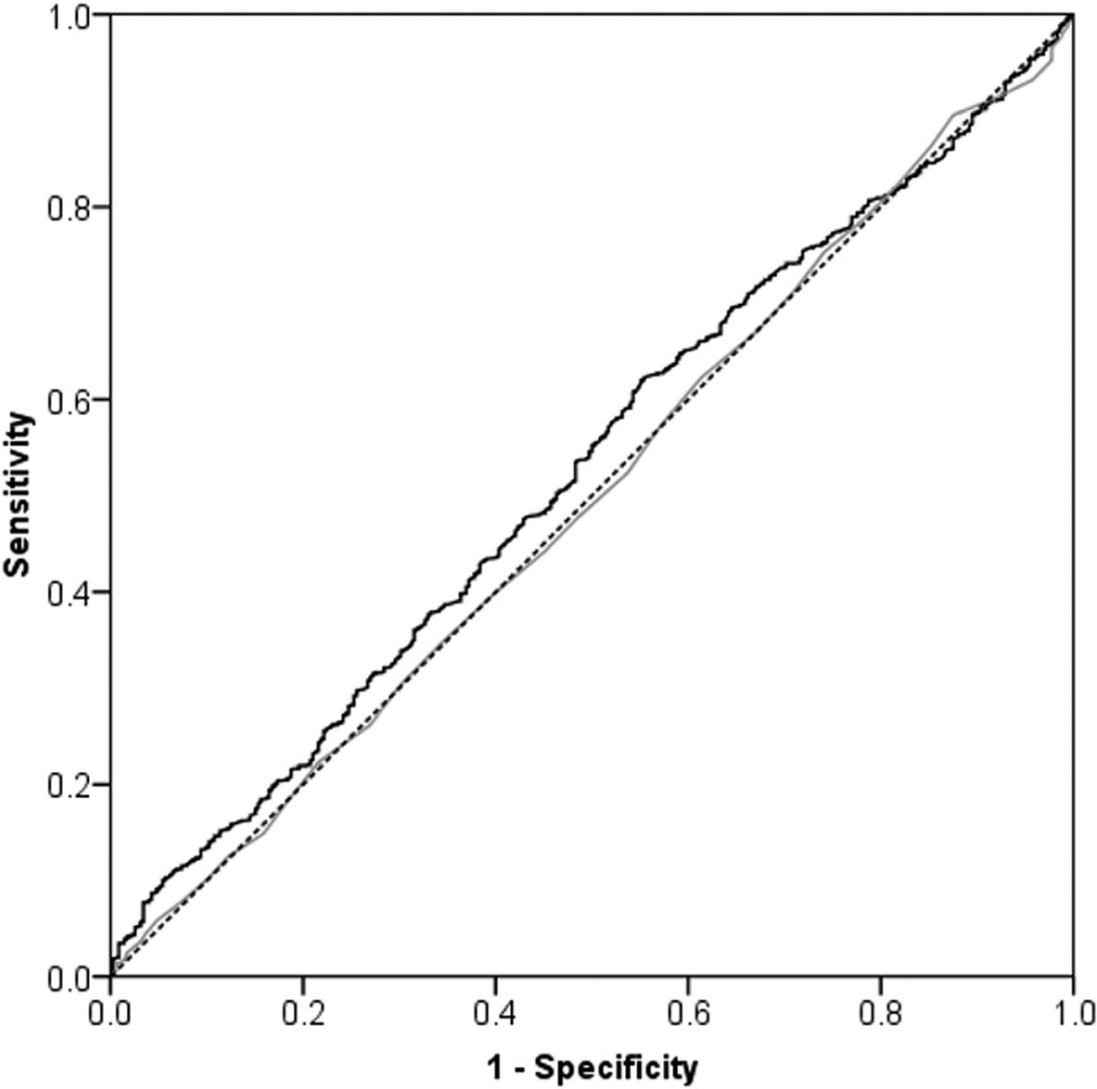
Receiver operating characteristic (ROC) curve for predicting a 5-point or greater (minimal clinically important difference (MCID)) improvement in the Oxford knee score (OKS) from 6 to 12 months according to body mass index (BMI) (black line) and preoperative OKS (grey line). Dashed line is a reference line for no predictive ability

There were 381 (24.2%) patients that had improvement in the OKS greater than the MCID from 6 to 12 months. However, in contrast 203 (12.9%) patients had a decrease in OKS greater than the MCID from 6 to 12 months. Patients with a change greater than the MCID from 6 to 12 months had a significantly worse 6-month OKS, which was significantly better at 12 months when compared to those who did not have a change greater than the MCID (Table 3). After adjusting for confounding, patients with a lower BMI, without diabetes mellitus, with a better preoperative OKS or a worse 6-month OKS were more likely to have an increase in their OKS from 6 to 12 months of greater than the MCID (Table 4). ROC curve analysis showed the 6-month OKS to be a moderately reliable predictor of a MCID improvement in the 6-month to 12-month OKS (Fig. 3), with a threshold value < 36 points being predictive (AUC 0.73, 95% CI 0.70–0.75, *p* < 0.001). In contrast, BMI (AUC 0.53, 95% CI 0.49–0.56, *p* = 0.111) and the preoperative OKS (AUC 0.50, 95% CI 0.47–0.54, *p* = 0.996) were not reliable predictors of achieving an improvement greater than the MCID (Fig. 4).

**Table 3:** Preoperative and postoperative OKS in patients with and without a change greater than the MCID from 6 to 12 months

OKS (mean, SD)	Change greater than MCID		Difference (95% CI)	***P*** value*
	Yes (*n* = 381)	No (*n* = 1193)		
**Preoperative**	21.1 (7.6)	21.3 (7.8)	0.2 (−0.7 to 1.1)	0.694
**6 Months postoperative**	30.4 (7.9)	36.7 (8.9)	6.2 (5.2 to 7.2)	< 0.001
**12 Months postoperative**	38.8 (7.4)	35.4 (10.1)	3.4 (2.3 to 4.5)	< 0.001

*OKS* Oxford knee score, *MCID* minimal clinically important difference

*unpaired *t* test

**Table 4:** Independent predictors of a MCID in the improvement in the OKS from 6 to 12 months after TKA

Demographic	Descriptive	Odds ratio	95% CI	***P*** value
**BMI**	For each BMI point	0.97	0.94 to 0.99	0.028
**Comorbidity**	Not present	Reference*		
	Diabetes mellitus	0.61	0.39 to 0.96	< 0.001
**Functional measure**				
**OKS**	Preoperative	1.05	1.02 to 1.07	< 0.001
	6 Months	0.91	0.89 to 0.92	< 0.001

Logistic regression analysis: entering all variables into the model (Table 1) using forward conditional methodology. The *R*^2^ value of the model was 0.10

*MCID* minimal clinically important difference, *OKS* Oxford Knee Score, *TKA* total knee arthroplasty, *BMI* body mass index, *CI* confidence interval

*Odds ratio relative to a patient without the stated comorbidity

## Discussion

This study has shown there was a statistically significant change in the OKS from 6 to 12 months after TKA, but this was not clinically significant when assessed as an overall mean change. Patients without diabetes mellitus, with a lower BMI, a better (greater) preoperative OKS, a worse 6-month OKS and a better EQ-5D at 6 months were associated with a greater improvement in the OKS from 6 to 12 months. Nearly a quarter of the patients had a clinically significant improvement in their OKS from 6 to 12 months. Absence of diabetes mellitus or having a 6-month OKS < 36 points was associated with a clinically significant improvement in the 6 to 12 month OKS.

The statistically significant mean improvement of 1 point demonstrated in the OKS between 6 and 12 months is a novel aspect of this study; however, it is smaller than the 2-point value reported by Browne et al. [7] in their systematic review. Brown et al. [7] compared the mean OKS at 6 months using outcome data taken from Health and Social Care Information Centre in England (*n* = 30,616) and compared this to 12-month data from 13 published cohort studies. These are non-linked data and may represent a skewed assessment of registry patients (no exclusion criteria) with cohort study data (specific inclusion criteria) that may have resulted in the conclusion of greater improvement. The current study used linked data (same patients) and should represent the real mean change in the OKS experienced by a patient between 6 and 12 months. The MCID in the OKS is 5 points [14] and therefore the 1-point change identified in the current study does not relate to a clinically perceived difference when assessing a cohort of patients between 6 and 12 months after their TKA.

The authors are not aware of a previous publication defining or suggesting the optimal timepoint after TKA when outcome should be assessed using the OKS. This is a fundamental aspect to any study using the OKS; be it a primary or secondary aim, the time of assessment should probably represent the maximal outcome achieved by the patient group being evaluated. The 6-month OKS will likely improve by more than 1 point at 12 months, but in nearly a quarter of patients the improvement will be clinically significant. The recent Total or Partial Knee Arthroplasty Trial (TOPKAT) study assessed the OKS at 5 years after knee arthroplasty as the primary end point, which was not clinically significantly different from that at 1 –5 years in the two groups [10]. This longer follow up of 1–5 years not only delays the results of the study but also adds to the overall cost of the study and potentially increases the loss to follow up. Studies reporting the OKS prior to 1 year, for example those using 6-month data from the National Joint Registry of England and Wales, may not represent the true potential postoperative OKS among patients and may lead to differing conclusions when compared to those studies using data from 1 year onwards. The authors suggest that the 1-year OKS should be used as the benchmark timepoint to assess the outcome of TKA, being the peak score when assessing a cohort of patients.

Despite the current study not demonstrating a clinically significant improvement in the OKS between 6 and 12 months, this does not necessarily mean a patient does not have a clinical improvement in their symptoms and may simply represent a limitation in the OKS to detect such a change. The OKS was originally designed by using feedback from patients waiting to have a TKA and therefore could be more reflective of the preoperative symptom [2]. In contrast, the Forgotten Joint score was designed to be reflective of the postoperative symptom state and Giesinger et al. [15] demonstrated a further improvement in scores between 1 and 2 years using this tool. Scott el al [8] described a cohort of patients undergoing TKA, who were followed up for 5 years, and found no significant change in the OKS between 1 and 5 years (1-point decrease), suggesting the clinical peak of the OKS after TKA is reached at 1 year.

The relatively short follow up at 1 year is a limitation of this study, as the OKS could potentially change beyond this timepoint. This was due to financial reasons at the study centre with cessation of routine functional outcome collection at this timepoint. Williams et al. [9] demonstrated that the maximal OKS was achieved at 1 year with a score of 34 points, which remained the same until the fifth postoperative year when the OKS fell to 33 points after which there was a gradual decline to 30 points at 10 years after TKA. The observed maximal peak in the OKS between 1 and 4 years demonstrated by Williams et al. [9] supports the results of the current study, with assessment of the peak functional outcome being achieved at 1 year after TKA. A further limitation was the retrospective design of the current study, which did not allow additional assessment of those patients achieving a clinically significant improvement in the OKS between 6 and 12 months. Factors associated with slower recovery during the first 6 months, such as postoperative stiffness or pain in other joints, may have been identified and accounted for in the analysis [16, 17]. Last, another limitation was that 38.3% of patients were excluded from the analysis due to incomplete OKS questionnaires.

## Conclusion

Overall, there was no clinically significant change in the OKS from 6 to 12 months; however, however a clinically significant improvement was observed in approximately a quarter of patients and was more likely among those scoring < 36 points at 6 months.

## Data Availability

The datasets analysed during the current study are not publicly available due ethical approval and patient anonymity.

## Abbreviations

BMI: Body mass index
COPD: Chronic obstructive airways disease
CI: Confidence interval
EQ: EuroQoL
EQ 5D: EuroQoL 5-Dimension questionnaire
MCID: Minimal clinically important difference
MI: Myocardial infarction
OKS: Oxford knee score
PROMs: Patient reported outcome measures
PVD: Peripheral vascular disease
SD: Standard deviation
TKA: Total knee arthroplasty

## References

[1] Collins NJ, Roos EM (2012) Patient-reported outcomes for total hip and knee arthroplasty. Commonly used instruments and attributes of a “good” measure. Clin Geriatr Med. 10.1016/j.cger.2012.05.007 10.1016/j.cger.2012.05.007 22840304

[2] Dawson J, Fitzpatrick R, Murray D, Carr A (1998) Questionnaire on the perceptions of patients about total knee replacement. J Bone Jt Surg - Ser B. 10.1302/0301-620X.80B1.7859 10.1302/0301-620x.80b1.7859 9460955

[3] Liddle AD, Pandit H, Judge A, Murray DW (2015) Patient-reported outcomes after total and unicompartmental knee arthroplasty: a study of 14 076 matched patients from the National Joint Registry for EngLand and Wales. Bone Jt J. 10.1302/0301-620X.97B6.35155 10.1302/0301-620X.97B6.35155 26033059

[4] Beard DJ, Harris K, Dawson J et al (2015) Meaningful changes for the Oxford hip and knee scores after joint replacement surgery. J Clin Epidemiol. 10.1016/j.jclinepi.2014.08.009 10.1016/j.jclinepi.2014.08.009 PMC4270450 25441700

[5] Clement ND, MacDonald D, Burnett R (2013) Predicting patient satisfaction using the Oxford knee score: where do we draw the line? Arch Orthop Trauma Surg. 10.1007/s00402-013-1728-3 10.1007/s00402-013-1728-3 23525559

[6] Clement ND, Burnett R (2013) Patient satisfaction after total knee arthroplasty is affected by their general physical well-being. Knee Surg Sport Traumatol Arthrosc. 10.1007/s00167-013-2523-y 10.1007/s00167-013-2523-y 23670127

[7] Browne JP, Bastaki H, Dawson J (2013) What is the optimal time point to assess patient-reported recovery after hip and knee replacement? A systematic review and analysis of routinely reported outcome data from the English patient-reported outcome measures programme. Health Qual Life Outcomes. 10.1186/1477-7525-11-128 10.1186/1477-7525-11-128 PMC3733605 23895227

[8] Scott CEH, Clement ND, MacDonald DJ et al (2015) Five-year survivorship and patient-reported outcome of the Triathlon single-radius total knee arthroplasty. Knee Surg Sport Traumatol Arthrosc. 10.1007/s00167-014-2922-8 10.1007/s00167-014-2922-8 24623184

[9] Williams DP, Blakey CM, Hadfield SG, Murray DW, Price AJ, Field RE (2013) Long-term trends in the Oxford knee score following total knee replacement. J Bone Jt Surg - Ser B. 10.1302/0301-620X.95B1.28573 10.1302/0301-620X.95B1.28573 23307672

[10] Beard DJ, Davies LJ, Cook JA et al (2019) The clinical and cost-effectiveness of total versus partial knee replacement in patients with medial compartment osteoarthritis (TOPKAT): 5-year outcomes of a randomised controlled trial. Lancet. 10.1016/S0140-6736(19)31281-4 10.1016/S0140-6736(19)31281-4 PMC6727069 31326135

[11] Baker PN, van der Meulen JH, Lewsey J, Gregg PJ (2007) The role of pain and function in determining patient satisfaction after total knee replacement. Data from the National Joint Registry for England and Wales. J Bone Jt Surg - Ser B. 10.1302/0301-620X.89B7.19091 10.1302/0301-620X.89B7.19091 17673581

[12] Burn E, Liddle AD, Hamilton TW et al (2018) Cost-effectiveness of unicompartmental compared with total knee replacement: a population-based study using data from the National Joint Registry for England and Wales. BMJ Open. 10.1136/bmjopen-2017-020977 10.1136/bmjopen-2017-020977 PMC5931302 29706598

[13] Herdman M, Gudex C, Lloyd A et al (2011) Development and preliminary testing of the new five-level version of EQ-5D (EQ-5D-5L). Qual Life Res. 10.1007/s11136-011-9903-x 10.1007/s11136-011-9903-x PMC3220807 21479777

[14] Clement ND, MacDonald D, Simpson AHRW (2014) The minimal clinically important difference in the Oxford knee score and Short Form 12 score after total knee arthroplasty. Knee Surg Sport Traumatol Arthrosc. 10.1007/s00167-013-2776-5 10.1007/s00167-013-2776-5 24253376

[15] Giesinger K, Hamilton DF, Jost B, Holzner B, Giesinger JM (2014) Comparative responsiveness of outcome measures for total knee arthroplasty. Osteoarthr Cartil. 10.1016/j.joca.2013.11.001 10.1016/j.joca.2013.11.001 PMC3988962 24262431

[16] Clement ND, Bardgett M, Weir D, Holland J, Deehan DJ (2019) Increased symptoms of stiffness 1 year after total knee arthroplasty are associated with a worse functional outcome and lower rate of patient satisfaction. Knee Surg Sport Traumatol Arthrosc. 10.1007/s00167-018-4979-2 10.1007/s00167-018-4979-2 PMC6435608 29748697

[17] Maxwell J, Niu J, Singh JA, Nevitt MC, Law LF, Felson D (2013) The influence of the contralateral knee prior to knee arthroplasty on post-arthroplasty function: the multicenter osteoarthritis study. J Bone Jt Surg - Ser A. 10.2106/JBJS.L.00267 10.2106/JBJS.L.00267 PMC3748984 23780536

